# Inferring Kangaroo Phylogeny from Incongruent Nuclear and Mitochondrial Genes

**DOI:** 10.1371/journal.pone.0057745

**Published:** 2013-02-22

**Authors:** Matthew J. Phillips, Dalal Haouchar, Renae C. Pratt, Gillian C. Gibb, Michael Bunce

**Affiliations:** 1 School of Earth, Environmental and Biological Sciences, Queensland University of Technology, Brisbane, Queensland, Australia; 2 School of Veterinary and Life Sciences, Murdoch University, Perth, Western Australia, Australia; 3 Research School of Biology, Australian National University, Canberra, Australian Capital Territory, Australia; 4 Institute of Agriculture and Environment, Massey University, Palmerston North, New Zealand; BiK-F Biodiversity and Climate Research Center, Germany

## Abstract

The marsupial genus *Macropus* includes three subgenera, the familiar large grazing kangaroos and wallaroos of *M. (Macropus)* and *M. (Osphranter)*, as well as the smaller mixed grazing/browsing wallabies of *M. (Notamacropus)*. A recent study of five concatenated nuclear genes recommended subsuming the predominantly browsing *Wallabia bicolor* (swamp wallaby) into *Macropus*. To further examine this proposal we sequenced partial mitochondrial genomes for kangaroos and wallabies. These sequences strongly favour the morphological placement of *W. bicolor* as sister to *Macropus*, although place *M. irma* (black-gloved wallaby) within *M. (Osphranter)* rather than as expected, with *M. (Notamacropus)*. Species tree estimation from separately analysed mitochondrial and nuclear genes favours retaining *Macropus* and *Wallabia* as separate genera. A simulation study finds that incomplete lineage sorting among nuclear genes is a plausible explanation for incongruence with the mitochondrial placement of *W. bicolor*, while mitochondrial introgression from a wallaroo into *M. irma* is the deepest such event identified in marsupials. Similar such coalescent simulations for interpreting gene tree conflicts will increase in both relevance and statistical power as species-level phylogenetics enters the genomic age. Ecological considerations in turn, hint at a role for selection in accelerating the fixation of introgressed or incompletely sorted loci. More generally the inclusion of the mitochondrial sequences substantially enhanced phylogenetic resolution. However, we caution that the evolutionary dynamics that enhance mitochondria as speciation indicators in the presence of incomplete lineage sorting may also render them especially susceptible to introgression.

## Introduction

The family Macropodidae includes more than 60 species of bipedal hopping kangaroos and wallabies living throughout Australia, New Guinea and surrounding islands. The family has Late Oligocene-Early Miocene rainforest origins and its diversification primarily coincides with subsequent aridification, during which woodland and grassland habitats expanded [1], [2]. The most iconic and species-rich group of macropodids to exploit these more open mesic to semi-arid habitats is the genus *Macropus*. The 13 extant species are divided into three subgenera, (i) *M. (Macropus)*, including the grey kangaroos, (ii) *M. (Osphranter)*, including the red kangaroo and wallaroos and (iii) the *M. (Notamacropus)* wallabies.

Body size and foraging ecology vary substantially among kangaroos and wallabies. Species are sexually size-dimorphic (e.g. mean adult body mass in the red kangaroo, *M. rufus* is 26 kg ♀/66 kg ♂ [3]), although foraging is broadly similar among the sexes. Sanson [4] characterised macropodid dental grades associated with foraging ecology, contrasting browsers of dicotyledonous plants with grazes feeding primarily on grasses. Predominance of grazing and larger adult body mass (averaged over males and females [3], [5]) distinguish *M. (Macropus)* (26–33 kg) and *M. (Osphranter)* (17–46 kg) from the smaller, typically mixed browsing/grazing *M. (Notamacropus)* (4–16 kg).

**Figure 1:**
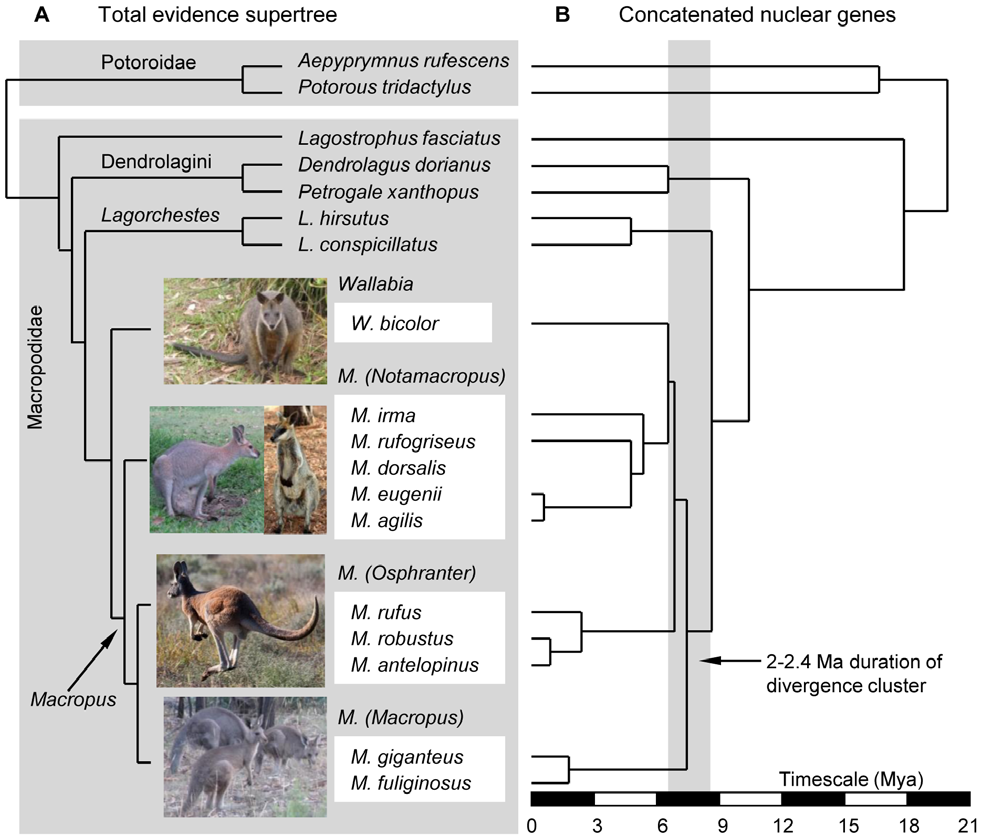
Phylogenetic relationships of *Wallabia* and the three *Macropus* subgenera, *M. (Macropus)*, *M. (Osphranter)* and *M. (Notamacropus)*. (A) The supertree of Cardillo et al. [6] summarizing previous molecular and morphological phylogenies and (B) Meredith et al.'s [7] evolutionary timescale (ave. of four BEAST analyses), showing the 2–2.4 Ma duration divergence cluster. Both trees are modified to include only the taxa sampled in the present study. Dendrolagini was not recovered by Cardillo et al. [6], however its inclusion in the summary tree is warranted on subsequent strong evidence from morphology [2] and all recent molecular analyses. Photos include (from the top) *W. bicolor*, *M. rufogriseus* (left), *M. irma* (right), *M. rufus and M. giganteus*. Photo credits – Matt Phillips, except *M. irma* (Ric Dawson) and *M. rufus* (Daniel Hoops).

Cardillo et al. [6] inferred a marsupial supertree from a comprehensive survey of earlier molecular and morphological phylogenies. This summary tree (modified in Figure 1A) closely matches the subsequent study by Meredith et al. [7], which sampled DNA sequences for five nuclear genes, including for 11 of the 13 *Macropus* species (Figure 1B). The two differences from the Figure 1A summary concern the relative affinities of the three *Macropus* subgenera and the placement of the monotypic *Wallabia bicolor*. In the former case, Meredith et al. [7] group *M. (Osphranter)* with *M. (Notamacropus)* to the exclusion of *M. (Macropus)*, though with weak support. The interrelations of these three subgenera have remained opaque to all data sources. Even consistent morphological support for grouping the larger *M. (Osphranter)* and *M. (Macropus)* hinges primarily on dental and palatal characters that may instead reflect correlations with grazing [8].

The more striking difference is Meredith et al. 's [7] placement of *W. bicolor* within *Macropus*, either as sister to *M. (Notamacropus)* or as sister to the more inclusive *M. (Notamacropus)*/*M. (Osphranter)* clade. On this basis the authors suggested subsuming *Wallabia* into *Macropus*, with subgeneric status for *M. (Wallabia)*. Many early workers [1], [9] also grouped *W. bicolor* with members of *M. (Notamacropus)* in the genus *Protemnodon* (which now includes only extinct members). Ride [10] however, noted that parallelism and plesiomorphy could explain anatomical similarities between *W. bicolor* and species now placed in *M. (Notamacropus)*, which overlap in size (*W. bicolor* adult mean, 15 kg [5]) and in foraging habits – although *W. bicolor* is more specialized for browsing. Morphological studies [2], [8], [11]–[13] and behavioural analysis [14] have since favoured placing *W. bicolor* outside of *Macropus*, although without identifying characters that provide unambiguous support.

Earlier molecular studies have been similarly indecisive on the relationship of *Wallabia* to *Macropus*. Analyses of allozymes [15] and mitochondrial 12/16SrRNA+tRNA-valine sequences [16], [17] favoured *Macropus* monophyly, to the exclusion of *W. bicolor*. Meanwhile, serology [18], microcomplement fixation [19] and DNA-DNA hybridization [20] tended to favour *W. bicolor* falling within *Macropus*, albeit often in different positions. Mitochondrial (mt) *Cytb* and nuclear *Selenocysteine tRNA* [21] did not clearly resolve affinities between the subgenera, while *Protamine P1* [22] favoured grouping *W. bicolor* with *M. rufogriseus*, leaving not only *Macropus*, but also *M. (Notamacropus)* paraphyletic.

This study expands the available 12S/16SrRNA and *Cytb* sequences and adds new *NADH1* and *NADH2* protein-coding sequences. Together, these provide a 5.6 kb mtDNA dataset for *W. bicolor*, nine *Macropus* species and seven outgroup macropodids and potoroids. The new sequences include the first molecular data for *M. dorsalis* and the first mtDNA for *M. irma*. Both of these wallabies are classified on morphology as members of *M. (Notamacropus)* [8].

We provide a more comprehensive examination of species relationships among kangaroos and wallabies by analysing the mtDNA alongside the five published nuclear genes (*BRCA1*, *IRBP*, *RAG1*, *ApoB* and *vWF*) from Meredith et al. [7]. Combining mt and nuclear sequences has previously provided strong statistical power for resolving family and ordinal-level marsupial relationships [23], [24]. However, concatenation is expected to mask uncertainty and potentially bias inference of relationships among closely diverged species, where multiple gene lineages persist through speciation events (incomplete lineage sorting, ILS) [25], [26].

We employ three “species tree” methods for combined analyses of the mt and nuclear genes in order to account for ILS among gene trees. The first of these, *BEAST [27] is highly parametric, employs the multi-species coalescent model and co-estimates the individual gene trees embedded within the species tree. The second, minimizing deep coalescences (MDC [28]) is a non-parametric alternative that uses a parsimony algorithm to identify the species tree requiring the fewest deep coalescent events among specified gene trees. The third species tree approach, Bayesian concordance analysis within BUCKy 1.4 [29] models gene tree incongruence while accounting for stochastic variation within posterior or bootstrap distributions of gene trees. Importantly, BUCKy does not assume any particular source of gene tree incongruence, unlike *BEAST and MDC, which both assume incongruence derives from ILS.

The potential importance of post-speciation gene flow in the present study is underlined by introgressive hybridization having been identified among natural populations of parapatric rock wallaby species (*Petrogale spp*. [30], [31]) and between the grey kangaroos, *M. giganteus* and *M. fuliginosus* [32]. Introgression however, can be difficult to distinguish from ILS [33], [34]. We use a simulation approach [35], [36] to distinguish these sources of incongruence.

The role of mtDNA for inferring relationships among closely related animal species has been much argued recently [37]–[41]. In several regards the mt genome should be an excellent marker. In contrast to the high rates of duplication and translocation of nuclear genes, the mt genome offers near-certain orthology for mammals, as long as appropriate practices are employed to avoid nuclear copies of mt genes [42]. Moreover, mitochondrial haploidy and uniparental inheritance confer ∼4-fold lower effective population size (*N*_e_) relative to nuclear DNA, such that mtDNA is expected to be a “leading indicator” of speciation [43]. Mitochondrial *N*_e_ and coalescent times may often be even further reduced by strong selection [37].

On the flip side of these arguments, population structure can diminish the influence of lower *N*_e_ on coalescence times [44]. Furthermore, the lack of recombination tends to lead genomes into fitness traps via a process known as Muller's ratchet [45]. Lower *N*_e_ and higher mutation rates can serve to accelerate this ratchet [46]. Resulting differences in mean fitness between populations and species can drive introgression of mtDNA, as demonstrated in *Drosophila* [47].

In this study we examine the utility of mtDNA for complementing nuclear sequences in reconstructing the phylogeny of kangaroos and wallabies. Inclusion of mtDNA substantially improves phylogenetic resolution of clades that have apparently been subject to incomplete lineage sorting among nuclear loci. In turn, it is encouraging that the nuclear signal overwhelms the mitochondrial signal where the latter is discordant with both the nuclear and morphological data.

## Materials and Methods

### Ethics Statement

DNA and tissue samples were obtained from pre-existing collections as donations from The Australian Centre for Ancient DNA (University of Adelaide), The Research School of Biology (Australian National University), The Department of Environment and Conservation, Western Australia and as loans from The Australian National Wildlife Collection, Canberra – in each case with permission from the relevant authorities within these institutions. One additional frozen tissue sample was purchased from a local butcher (EcoMeats) in Canberra. No live animals were sampled and none of the DNA/tissue collection or handling procedures required either approval or a permit from a review board or ethics committee. Sequences published previously by other groups were obtained from GenBank.

### Taxon sampling and DNA sequencing

In order to reconstruct a mitochondrial tree for kangaroos and wallabies we targeted three protein-coding genes, *NADH1*, *NADH2* and *Cytb* along with the 12S and 16S ribosomal RNA genes. Taxon sampling focused on ten *Macropus* species and the monotypic *Wallabia bicolor*. The affinities of the three extant *Macropus* species not included here are uncontroversial [6], [8], [48] and add little to the sampled diversity. *M. parma* and *M. parryi* are nested within *M. (Notamacropus)* and *M. bernardus* groups with the other wallaroos within *M. (Osphranter)*. Outgroup sampling includes the macropodids, *Lagorchestes (Lagor. hirsutus* and *Lagor. conspicillatus*), Dendrolagini (*Petrogale xanthopus* and *Dendrolagus dorianus*) and *Lagostrophus fasciatus*, in addition to the potoroids, *Aepyprymnus rufescens* and *Potorous tridactylus*.

Mitochondrial DNA was sequenced from DNA previously extracted at University of Adelaide (*A. rufescens*, *D. dorianus*, *P. xanthopus*, *Lagor. conspicillatus*, *W. bicolor* and *M. fuliginosus*) and Australian National University (*M. eugenii* and *M. rufogriseus*). For the remaining new sequences, DNA was extracted from tissue samples. These were provided by collections at Murdoch University (*M. rufus*), The Department of Environment and Conservation, WA (*M. irma*) and The Australian National Wildlife Collection (*M. dorsalis*) or purchased from EcoMeats in Canberra (*M. giganteus*). In addition, we sequenced two nuclear genes (*IRBP* and *ApoB*) from *M. irma* and *W. bicolor* to validate the provenance of our samples, given that their mtDNA placements differed from Meredith et al. [7]. Using our *IRBP* and *ApoB* sequences in place of Meredith et al. 's varied the maximum likelihood bootstrap support on the nuclear data for the placements of *M. irma* and *W. bicolor* by <1.5%. As a default however, we preferentially use the previously available *W. bicolor* sequences, which derive from the same individual as each of the other nuclear loci. For *M. irma* we use our *IRBP* and *ApoB* sequences, which cover a 28 bp sequencing gap and resolve for six ambiguity codes in the previously available sequences.

DNA extraction for *M. rufus* and *M. irma* was carried out at Murdoch University using a Qiagen DNeasy kit (Qiagen Sciences, MD, USA) and at Australian National University for all other taxa, using the salting out method (following [49]). DNA was amplified using standard PCR protocols on a Corbet Research iPAQ thermocycler (NSW, Australia). Primers and amplification conditions are provided in Table S1. All amplicons were sequenced by Macrogen (Seoul, South Korea). Nuclear sequences have been submitted to GenBank for *M. irma* (*IRBP*; JN967008, *ApoB*; JN967009) and for *W. bicolor* (*IRBP*; KC429577, *ApoB*; KC429578). GenBank accession details for the mt sequences are provided in Table S2.

### Data matrices

**Figure 2:**
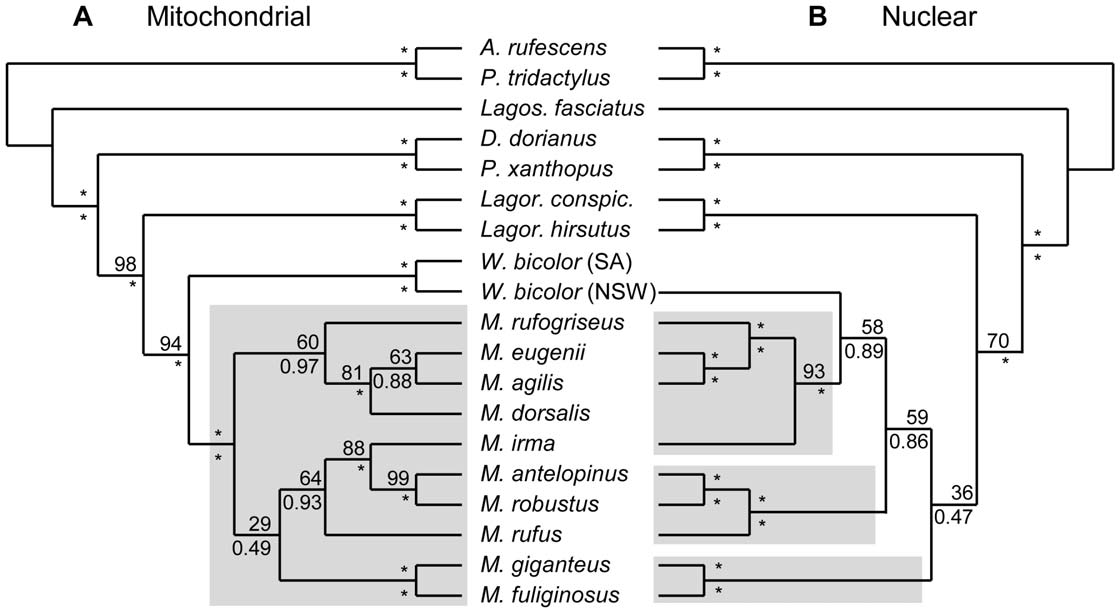
Phylogenetic analysis of kangaroos and wallabies. Maximum likelihood phylogenies inferred from the (A) mitochondrial (Mt_16_) and (B) nuclear (Nuc_17_) concatenated datasets, with RAxML bootstrap values (BP_ML_) above branches and MrBayes Bayesian posterior probabilities (BPP) below branches. The mt placement of *M. dorsalis* is derived from the reduced-length Mt_17_ and the mt placements of *M. antilopinus* and *W. bicolor* (NSW, New South Wales) are derived from the *Cytb*_18_ alignment. Support for grouping *M. eugenii* and *M. agilis* increases (BP_ML_ = 88; BPP = 0.98) for Mt_16_, which excludes *M. dorsalis*, but increases sequence length. Asterisks indicate full support. Clades including members of *Macropus* are shaded.

The primary mitochondrial dataset (Mt_16_) combines the *NADH1*, *NADH2* and *Cytb* protein-coding genes with the 12S and 16S rRNA genes. The sequences were initially aligned in ClustalW2 [50] with penalties of 5 for gap opening and 0.2 for gap extension. Manual adjustments were then made in Se-Al 2.0a [51], where sites with ambiguous homology were excluded, leaving a 5,593 bp mtDNA alignment. An expanded matrix (Mt_17_) includes *M. dorsalis*; although poor tissue preservation resulted in low DNA yields and the sequence is 72% complete, missing 147 bp of *NADH2*, all of *Cytb* and 269 bp of the rRNA genes. The only mt sequence available on GenBank for *M. antilopinus* was included in a 1,146 bp *Cytb*_18_ matrix (with the taxa from Mt_16_ and an additional *W*. *bicolor* sequence) and was sufficient to confidently place *M. antilopinus* as sister to *M. robustus*, in agreement with nuclear genes (Figure 2).

Previous research has demonstrated a requirement to ameliorate nucleotide compositional biases among marsupial mt genomes for phylogenetic inference of ordinal level relationships [23], [24]. By contrast, the present focus on closely related genera is relatively shallow. Our composition homogeneity χ^2^ testing on Mt_16_ in PAUP* 4.0b10 [52] with uninformative and gapped sites excluded offers little evidence for base compositional non-stationarity among the *Macropus* and *Wallabia* ingroup (protein 1^st^ codons: P = 0.4222, 2^nd^ codons: P = 0.9708, 3^rd^ codons: P = 0.5810, RNA stems: P = 0.9941, RNA loops: P = 0.3400).

We analyse the mt sequences alongside the nuclear dataset of Meredith et al. [7], which includes protein-coding segments from *BRCA1* (exon 11, breast and ovarian cancer susceptibility gene), *ApoB* (exon 26, Apolipoprotein B), *IRBP* (exon 1, interphotoreceptor retinoid binding protein gene), *RAG1* (intronless recombination activating gene-1) and *vWF* (exon 28; vonWillebrand factor gene). Aligning the nuclear sequences followed the procedure described above for the mtDNA. Two 5,988bp matrices were constructed, Nuc_16_, with taxon sampling matching Mt_16_ and also Nuc_17_, which further includes *M. antilopinus*. Combined analyses (MtNuc_16_) concatenated the Mt_16_ and Nuc_16_ matrices.

### Phylogenetic inference of mitochondrial and nuclear gene trees

Kangaroo phylogeny was inferred under maximum likelihood (ML) and Bayesian inference from the mitochondrial and nuclear sequences separately and concatenated, as well as for the individual nuclear genes. Substitution model categories for each data partition employed the more general of the jModelTest 0.1.1 [53] hLRT or AIC recommendations (Table S3) or the next most general available for each phylogenetic inference program. Substitution was modelled separately among the mt protein-coding codon positions and RNA stem and loop sites.

Our initial efforts to reconstruct kangaroo phylogeny employed Bayesian inference in MrBayes 3.1.2 [54] and ML in RAxML vGUI093 [55]. MrBayes analyses ran two independent sets of two MCMC chains for 6,000,000 (Nuc_17_, MtNuc_16_) or 4,000,000 (Mt_16_, Mt_17_, *Cytb*_18_ and individual nuclear genes) generations, with trees sampled every 2,500 generations. Burn-ins varied from 500,000 to 1,200,000 generations, and were chosen to ensure that –ln*L* had plateaued, clade frequencies had converged between runs and estimated sample sizes for substitution parameters were >200 (using Tracer v1.5 [56]). ML analyses in RAxML carried out 500 full bootstrap replicates. Branch-length multipliers and substitution models were partitioned among protein codons and RNA stems and loops for each of the ML and Bayesian analyses, with MtNuc_16_ further partitioned between mt and nuclear sites.

Support among alternative topologies was further examined with the approximately unbiased (AU) test [57], using the RELL method (100,000 replications) within CONSEL [58]. Site likelihoods employed in CONSEL were inferred in PAUP*, with all substitution parameters and branch-lengths ML optimized separately for each of the protein codon and RNA structural partitions, for each tree hypothesis. Maximum likelihood trees conforming to the alternative Mt_16_ and Nuc_17_ placements of *W. bicolor* and *M. irma* (Figure 2) were identified for each gene in PAUP* with 20 random addition heuristic searches. Support among these individual genes for the alternative placements was compared with SH tests [59], which as pairwise comparisons reduce to equivalency with AU and KH [60] tests. ML bootstrapping (500 replicates) for each gene was also performed in PAUP* with the substitution model parameters estimated in the earlier heuristic searches.

We estimated a mitochondrial timescale for kangaroo evolution using BEAST v.1.6.1 [61] with Mt_16_ partitioned as per the phylogenetic analyses. An uncorrelated relaxed clock model was used with rates among branches distributed according to a lognormal distribution. Note that likelihood ratio tests in PAUP* rejected strict clocks for both the mt and nuclear sequences (P<0.01). Four independent runs totalling 40,000,000 MCMC generations ensured estimated sample size values >100 (as estimated in Tracer v1.5) for all node height, prior, posterior, −ln*L*, tree, and substitution parameters. Chains were sampled every 5,000 th generation after burn-ins of 1,000,000 generations.

Four fossil-based priors were used to calibrate the BEAST analysis. (i) Potoroidae/Macropodidae (15.97–28.4 Ma), with the minimum based on the Early Miocene macropodid, *Ganguroo* [2] and the maximum covering putative Late Oligocene macropodids [62] and potoroids [63]. (ii) Macropodidae (11.6–23 Ma), with the Middle Miocene macropodid, *Wanburoo* [2] providing the minimum bound and the maximum acknowledging that earliest Miocene macropods fall outside of the macropodid crown. *Ganguroo* is also a candidate for calibrating Macropodidae, however, its placement within this crown clade is not well resolved [2]. (iii) Dendrolagini (4.46–16.0 Ma), with the minimum based on the Hamilton fauna *Dendrolagus* [64] and the maximum bound recognising that all middle Miocene macropods fall outside the Dendrolagini crown. (v) *Macropus*/*Lagorchestes* (4.46–16.0 Ma), with the minimum based on Hamilton fauna *Macropus* [2] and the maximum bound recognising that all middle Miocene macropods fall outside of this crown clade.

Palaeontological data do not clearly favour any particular timing within the given bounds for calibrations (i), (iii) and (iv) and hence, flat priors were employed. A normal prior was employed for calibration (ii), in line with the recommendation of Ho and Phillips [65] for when the balance of evidence [2], [7], [62] suggests crown divergences fall well within the bounds. The normal prior was applied conservatively (90% of prior probability between the bounds).

### Partition homogeneity testing

To identify incongruence between partitions we performed the incongruence length difference test [66] in PAUP*. This test however, can be biased in cases where parsimony is statistically inconsistent [67]. To overcome this concern we also perform likelihood-based parametric bootstrap tests. For these we infer one ML score (ML_F_) with branch lengths and the models (as shown in Table S3) partitioned across genes, but assuming a single topology (T*) and another ML score (ML_V_) for which the topology is allowed to vary across genes. The difference between ML_F_ and ML_V_ provides a critical value for testing the null hypothesis that all genes evolved on the same phylogeny. Next we used Seq-Gen 1.3.2 [68] to simulate 200 datasets partitioned into the original mtDNA and five nuclear gene sequence lengths and evolved on topology T* with the original branch lengths and model parameters for each gene. The distribution of ML_F_ - ML_V_ differences from the 200 simulated datasets was then compared with the critical value from the original dataset.

### Species tree reconstruction

*BEAST analysis [27] within BEAST v1.6.1 employed the multi-species coalescent to infer the species tree underlying the mt and five nuclear gene trees coestimated from MtNuc_16_. The mtDNA was further partitioned into protein codon positions and RNA stems and loops for substitution modelling. Separate mt and nuclear uncorrelated lognormal relaxed clock models were used, with a Yule species tree prior and differential ploidy (autosomal nuclear and haploid mitochondrial). Eight independent runs totalling 80,000,000 MCMC generations ensured estimated sample size values >100 (estimated in Tracer v1.5) for all node height, prior, posterior, −ln*L*, tree, and substitution parameters. Chains were sampled every 5,000 th generation after burn-ins of between 1,000,000 and 4,000,000 generations. Given our focus on phylogeny rather than dating, we did not calibrate the *BEAST analysis, and so avoid potentially misleading influences of calibration priors on clade posterior probabilities [65]. Instead we provided a nominal mean substitution rate of 1.00 with unspecified time units for the nuclear data and allowed the mt rate to vary relative to this.

Minimizing deep coalescences, MDC [28] trees were inferred under the dynamic programming mode in PhyloNet 2.4 [69] from the mtDNA and five nuclear gene trees (Figure 2A, Figure S1), which were each estimated under ML bootstrapping in PAUP*. We collapsed branches that received <50% bootstrap support in these source trees to reduce the influence of stochastic artefacts among the individual genes on MDC tree building.

Bayesian concordance analysis within BUCKy [29] was run on 500 bootstrap replicate trees inferred in RAxML, for each of the six loci. Bootstrap distributions typically reflect stochastic variation in the gene tree estimates more closely than do Bayesian posterior distributions [24], [70]. Otherwise, BUCKy analyses employed default parameters, except where stated.

### Coalescent simulation

In order to better understand whether ILS could plausibly account for incongruence among gene trees we simulated the evolution of the five nuclear genes and the mtDNA under a coalescent process in MCcoal [71] within BPP 2.1 [72]. Two alternative guide trees were used for MCcoal, the combined data and nuclear-only *BEAST species trees. In the former case *M. irma* was excluded from the *BEAST analysis because of the concern that its mtDNA derives from introgression (see Discussion), which violates the assumptions of *BEAST. Instead, *M. irma* was grafted onto the tree – its temporal placement along the stem lineage from the other *Notamacropus* members was scaled in proportion to the nuclear-only *BEAST tree. For comparability the combined data and nuclear-only guide trees were both scaled to a root height of 20 Ma, closely matching both the mt BEAST estimate (21.3 Ma) and Meredith et al. 's [7] estimates from the concatenated nuclear genes (ave. BEAST estimate, 20.0 Ma).

**Figure 3:**
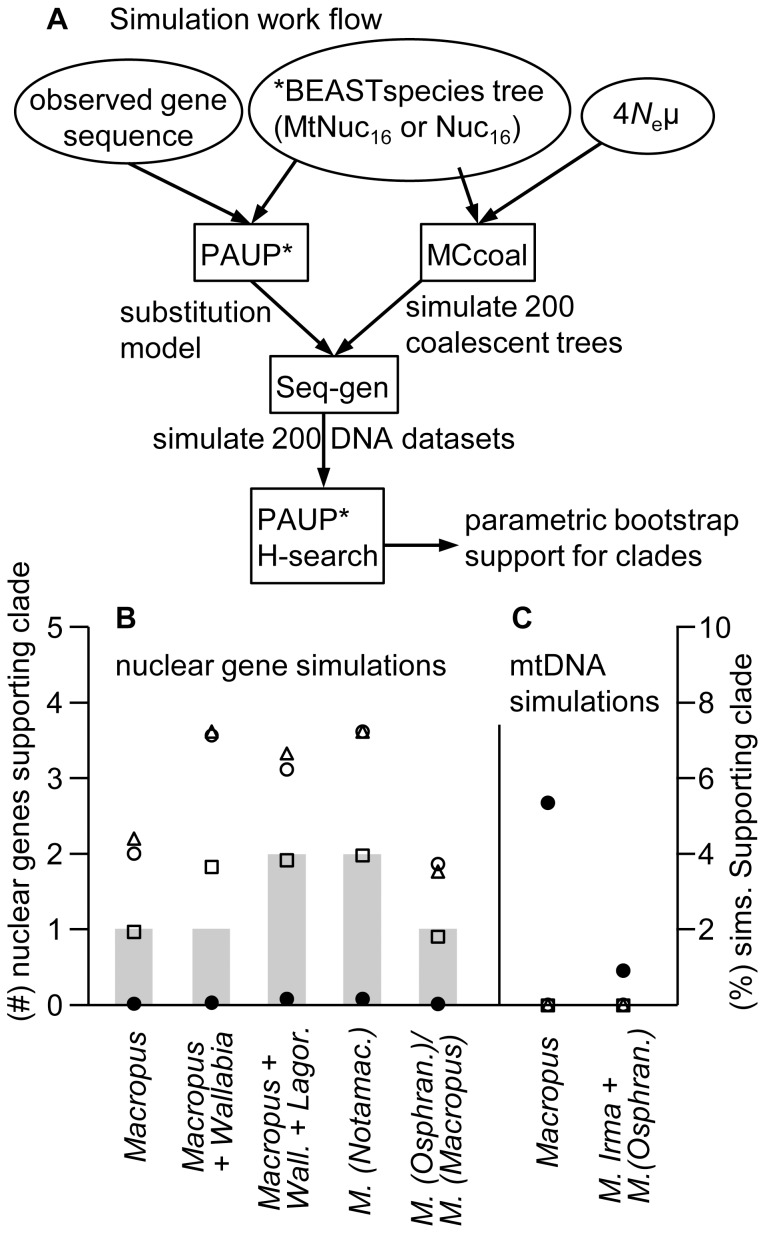
Macropodid clade support from datasets simulated under coalescence. (A) Simulation workflow. (B) Mean number of the five nuclear genes supporting each clade in maximum likelihood analyses of 200 simulations of the combined data *BEAST species tree for *N*_e_ values of 1,000 (triangle), 10,000 (open circle), 100,000 (square) and 1,000,000 (filled circle). For comparison, the grey bars show the number of genes supporting each clade on the observed data. (C) Percentage of ML analyses supporting each clade among 200 mtDNA simulations on the nuclear-only *BEAST species tree for *N*_e_ values set to mitochondrial equivalency for the same populations (one quarter of the corresponding nuclear values). Abbreviations: *Lagor*.; *Lagorchestes*, *Wall*.; *Wallabia*, *M. (Notamac.)*; *M. (Notamacropus)*, *M. (Osphran.)*; *M. (Osphranter)*.

The model that MCcoal simulates under (JC+Γ) is less complex than the models selected in jModelTest for each locus. Therefore we used a two-step simulation process (illustrated in Figure 3A). First, MCcoal was run on the species guide tree to provide simulated coalescent trees. Gene sequences were then simulated on these coalescent trees under their respective substitution models (Table S3) in Seq-Gen. All model parameters were estimated from the original data and the simulations maintained the aligned sequence length for the mtDNA and each nuclear gene.

MCcoal requires a population dynamics parameter θ = 4*N*_e_μ (2*N*_e_μ for mtDNA), where *N*_e_ is the effective population size and μ is the mutation rate per site per generation. Mutation rates per site per year were obtained by scaling PAUP* ML treelengths for each locus to the *BEAST timetree length. Generation time across macropods is not well studied, but we used an average of 7 years in consideration of life history data from most macropodid species [73]. The influence of effective population size was evaluated with *N*_e_ varied from 1,000 to 1,000,000.

## Results

### Phylogenetic inference from separate mitochondrial and nuclear sequences

**Figure 4:**
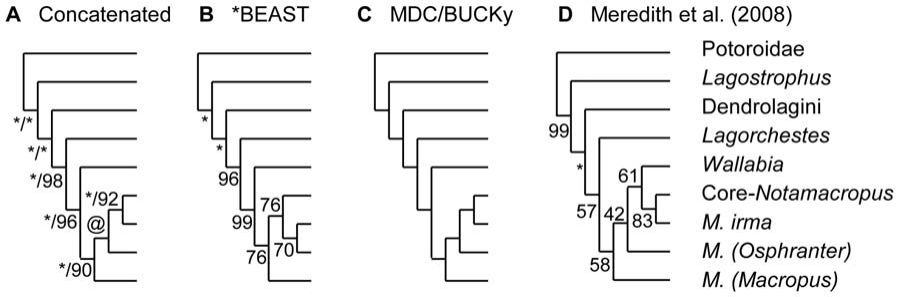
Macropodid species tree estimates from the combined mitochondrial and nuclear sequences (MtNuc_16_). (A) concatenated sequences, showing BPP/BP_ML_ (@ = 0.89/59), (B) *BEAST partitioned between the mtDNA and five nuclear genes, showing BPP values. (C) both MDC and BUCKy, which recovered the same tree from the mt and five nuclear gene trees. (D) Meredith et al. (2008) with BP_ML_ values included for comparison. Several supraspecific clades that were identical across all reconstructions were collapsed for visualization convenience. Relationships within each of the collapsed clades were as inferred in Figure 2. Asterisks indicate full BPP or BP_ML_ support.

Our analyses of the mt and nuclear sequences agree on grouping *Macropus* and *Wallabia* to the exclusion of the consecutive outgroups, *Lagorchestes*, Dendrolagini, *Lagostrophus* and Potoroidae (Figure 2). The inclusion of the mtDNA greatly enhanced phylogenetic resolution. Whereas four clades received 36–70% ML bootstrap support on the nuclear data alone, on the combined data all but one clade received ≥90% ML bootstrap support (Figure 4A). Only the relative affinities of the three *Macropus* sub-genera (*Macropus*, *Notamacropus* and *Osphranter*) remained poorly resolved. However, the combined result hides conflict between the mt (Figure 2A) and nuclear (Figure 2B) trees for the placements of *M. irma* and *W. bicolor*.

The nuclear data favours *M. irma* and *W. bicolor* as consecutive sister groups to the wallabies we refer to as core-*Notamacropus*, which here includes *M. rufogriseus*, *M. eugenii*, *M. agilis* and *M. dorsalis*. Placing *M. irma* with core-*Notamacropus* receives high ML bootstrap support (BP_ML_, 93%) and Bayesian posterior probability (BPP, 1.00). Further expanding this clade to include *Wallabia* is only supported modestly (BP_ML_ 58%, BPP 0.89). These results closely mirror Meredith et al. [7]. Our mitochondrial trees strongly conflict with these placements, instead favouring *M. irma* as sister to the wallaroos (*M. robustus*, *M. antilopinus*) (BP_ML_ 88%, BPP 1.00) and placing *W. bicolor* outside a monophyletic *Macropus* (BP_ML_ 100%, BPP 1.00).

Maximum likelihood AU testing reveals strong incongruence between the nuclear and mt data for the placements of both *W. bicolor* and *M. irma*. Table 1A shows that for Mt_16_ the favoured nuclear placement for *W. bicolor* as sister to the subgenus *M. (Notamacropus)* is rejected at P = 0.008 and reciprocally, AU testing on Nuc_17_ rejects the favoured mt placement for *W. bicolor* as sister to all *Macropus* at P = 0.011. Similarly for *M. irma* (Table 1B), the favoured mt placement as sister to *M. robustus* is rejected with the nuclear data (P<0.001) and reciprocally, the favoured nuclear placement as sister to core-*Notamacropus* is rejected with the mtDNA (P = 0.031).

**Table 1:** Approximately unbiased (AU) test results.

	Mitochondrial genes		Nuclear genes	
	–*lnL*	P-value	–*lnL*	P-value
(A) Placement of *Wallabia*				
1. Sister to *Macropus*	25,140.45	best	+20.06	0.011
2. Sister to *M. (Notamacropus)*	+31.505	0.008	13,107.34	best
				
(B) Placement of *M. irma*				
1. Sister to *M. (Notamacropus)*	+16.90	0.031	13,107.34	best
2. Sister to *M. robustus*	25,140.45	best	+90.16	<0.001
3. Sister to *M. (Osphranter)*	+6.21	0.157	+24.14	0.063
				
(C) Macropus subgenera relative affinities ˆ				
1. *M. (Notamacropus)*+*M. (Osphranter)*	+1.39	0.317	13,107.34	best
2. *M. (Osphranter)*+*M. (Macropus)*	25,140.45	best	+12.95	0.052
3. *M. (Macropus)*+*M. (Notamacropus)*	+0.62	0.455	+11.39	0.161
				
(D) Placement of *Lagorchestes*				
1. Sister to *Wallabia*+*Macropus*	25,140.45	best	13,107.34	best
2. Sister to *Wallabia*	+8.59	0.038	+25.82	0.009
3. With *Macropus*	+7.55	0.103	+0.94	0.462
				
(E) Placement of *M. dorsalis* (5 highest)				
1. Sister to (*M. agilis*+*M. eugenii*)	19,771.52	best		
2. Sister to *M. agilis*	+4.47	0.334		
3. Sister to *M. eugenii*	+7.17	0.079		
4. Sister to all other *M. (Notamacropus)* #	+10.25	0.134		
5. Sister to all other *Macropus*	+13.35	0.098		

Nuclear sequences are partitioned into protein codon positions and mitochondrial sequences are partitioned into protein codon positions and RNA stems and loops.

Comparisons (A) – (D) employ Mt_16_ and Nuc_17_. Comparison (E) employs Mt_17_.

ˆ Allowing *W. bicolor* and *M. irma* to float unconstrained on the tree

# Not including *M. irma*, which is favoured as sister to *M. robustus* on the mt data.

Turning to the individual nuclear genes, support for the favoured mt versus nuclear placements reveals distinctly different patterns for *M. irma* and *W. bicolor* (Table 2). All of the nuclear genes favour an *M. irma* relationship with *M. (Notamacropus)* over the mt relationship with *M. (Osphranter)* – except *BRCA1*, for which both of these relationships were equally likely. In contrast, the overall nuclear placement of *W. bicolor* as sister to *M. (Notamacropus)* is only favoured over the mt placement by *BRCA1* and *vWF*. Another gene (*IRBP*) instead favours the mt placement of *W. bicolor* outside *Macropus*, while the ML analyses for *ApoB* and *RAG1* find the nuclear and mt hypotheses for *W. bicolor* affinities to be equally likely.

**Table 2:** Individual nuclear gene –ln*L* differences and SH test results.

	*IRBP*	*vWF*	*ApoB*	*BRCA1*	*RAG1*
(A) Placement of *W. bicolor*					
1. Outside monophyletic *Macropus*	**2765.74**	+10.18	1763.63	+17.45	1008.98
2. With *M. (Notamacropus)*	+7.95	**2057.71**	1763.63	**5488.65**	1008.98
	P = 0.117	P = 0.070	—	P = 0.055	—
(B) Placement of *M. irma*					
1. With *M. (Osphranter)*	+14.10	+10.18	+2.85	5504.78	+0.05
2. With *M. (Notamacropus)*	**2763.58**	**2057.71**	**1763.82**	5504.78	**1009.18**
	P = 0.045	P = 0.042	P = 0.226	—	P = 0.646

ML placements in bold.

To ensure relevance of the individual gene results to the overall nuclear phylogeny, the relative positions of the outgroups and placements within *M. (Macropus)*, *M. (Osphranter)* and core-*Notamacropus* were fixed (see Figure 2).

Our analyses of Mt_17_ show that *M. dorsalis* groups with *M. eugenii* and *M. agilis* (BP_ML_ = 81%, BPP = 1.00; Figure 2A), with the latter two wallabies favoured as sister taxa (BP_ML_ = 63%, BPP = 0.88). AU testing (Table 1E) echoes these results, favouring *M. dorsalis* as sister to M. *eugenii* and *M. agilis*, although with other placements of *M. dorsalis* within core-*Notamacropus* rejected only at modest significance levels (P values from 0.079–0.334).

### Kangaroo species tree inference

Partition homogeneity testing performed in PAUP* identified significant incongruence between the mt and nuclear datasets (P = 0.027) and between the five nuclear genes (P = 0.003). Nuclear gene trees are shown in Figure S1. These parsimony-based results are in agreement with the likelihood-based parametric bootstrap test. For the latter, the improvement in likelihood of partitioning over concatenation for the mt and nuclear sequences (MtNuc_16_) and among the five nuclear genes (Nuc_16_) was 30.44 and 82.23 –ln*L* units respectively. In both cases these critical values fall higher than the distribution of likelihood improvements from partitioning for each of the 200 simulated datasets, therefore rejecting homogeneity at P<0.005.

We employed four approaches to inferring the kangaroo species tree from the mtDNA and five nuclear genes. First the data were concatenated, with substitution models and relative rates partitioned between mt and nuclear sequences and within these, between the protein-coding codon positions and RNA stems and loops. The concatenated MtNuc_16_ ML and Bayesian analyses provide a well resolved tree (Figure 4A) that combines the mitochondrial placement of *Wallabia* as sister to *Macropus*, with relationships among the *Macropus* species following the nuclear tree.

The second approach using MtNuc_16_ applied the multi-species coalescent within *BEAST to allow for ILS among the mtDNA and the five nuclear genes. As shown in Figure 4B *BEAST reconstructed the same topology as the concatenated analysis, except with *M. irma* as sister to *M. (Osphranter)* rather than core-*Notamacropus*. We also ran the *BEAST analysis without the putative introgressive hybrid, *M. irma*. *Macropus* monophyly was retained (BPP = 0.99), although among the subgenera, *M. (Osphanter)* grouped (at BPP = 0.86) with *M. (Macropus)*, instead of with *M. (Notamacropus)*.

Among the two other species tree approaches, BUCKy carries out Bayesian concordance analysis, which requires a prior level of discordance (α) to be assigned. We ran separate analyses with α at 0.5, 1, 5 and 10, which provide for a range of prior expectations for the 6 loci representing one or two distinct trees up to representing five or six distinct trees. The same concordance tree was recovered under each of these levels and shares the same topology with both the concatenated analysis (Figure 4A) and the MDC tree (Figure 4C). On the nuclear data alone, each of the species tree methods followed the concatenated nuclear tree (Figure 2B), in placing *W. bicolor* with *M. (Notamacropus)*. However, all methods used to combine the mt and nuclear sequences support *Macropus* monophyly.

### Coalescent simulations

There is agreement between the mtDNA and the majority of nuclear genes on deep and shallow clades with stem lineages ≥1.5 Ma on the combined species tree. Almost all of the discordance arises among a tight cluster of six consecutive divergences covering 4.2–4.9 Ma (see Table 3B,C for divergences) from the Macropodini/Dendrolagini divergence up to the *M. (Notamacropus)* crown divergence. In fact most gene discordance within this cluster (Figure S1) and the lowest bootstrap support (<60%, Figure 2B) involves the sequential divergence of *Lagorchestes, Wallabia* and each of the three *Macropus* subgenera covering just 2.0–2.4 Ma (Table 3; Figure 1B, grey strip). Partition homogeneity testing and AU testing (Tables 1,2) suggest that the extent of the incongruence cannot be explained by stochastic error alone. However, another explanation, incomplete lineage sorting is also consistent with the association between short stem length and incongruence.

**Table 3:** Macropodoid divergence time estimates in millions of years before present.

Clade	(A) mtDNA		(B) NucDNA	(C) MtNuc_16_ *BEAST
	Median	95%HPD	Meredith et al. [7]	Species tree
1. Macropodoidea	21.3	(16.0–26.8)	20.0	20.0
2. Potoroidae	16.5	(10.8–23.1)	16.4	17.0
3. Macropodidae	16.2	(12.2–20.6)	17.7	14.4
4. Dendrolagini/Macropodini	11.0	(8.2–14.0)	10.7	7.6
5. *Lagorchestes*/*Macropus*/*Wallabia*	9.7	(7.8–12.6)	8.8	6.6
6. *Macropus*/*Wallabia*	8.9	(6.6–11.5)	7.3	5.3
7. *Macropus*	7.6	(5.5–9.8)	—	4.8
8. *M. (Macropus)*/*M. (Osphranter)*	7.3	(5.3–9.5)	—	4.4
9. *M. irma*/*M. robustus*	5.9	(4.3–8.0)	—	
10. *Macropus*/*Wallabia* except *M*. *(Macropus)*	—	—	6.8	
11. *M. (Notamacropus)*/*Wallabia*	—	—	6.7	
12. *M. (Notamacropus)*	—	—	5.8	3.4

(A) BEAST analysis of Mt_16_, (B) average of four BEAST analyses on the five nuclear gene concatenate from Meredith et al. [7] and (C) *BEAST species tree analysis of MtNuc_16_.

Simulating the coalescent process over the combined data species tree allows inference of whether ILS provides a plausible explanation for the strong incongruence between the nuclear genes. In Figure 3B the grey bars show the number of nuclear genes supporting each of five short-stem-lineage clades within the diversification cluster. Phylogenetic analyses of the simulated datasets provide estimates for the probability of each gene supporting a given clade for alternative values of *N*_e_. The sum of these values over the five genes is the mean expectation for the number of genes supporting each clade. Coalescent time is very short with *N*_e_ = 1,000 and as a result the coalescent simulations overestimate the number of genes supporting each of the five clades. Under this scenario ILS appears to be a poor explanation for the lack of concordance among the nuclear genes. Increasing *N*_e_ to 10,000 makes little difference.

The greater potential for ILS with *N*_e_ = 100,000 provides for a remarkably close match to the observed gene support among the clades (Figure 3B). The sum of squares difference between expected and observed support for the five clades decreases from 13.25 and 12.20 for *N*_e_ = 1,000 and 10,000 respectively, to 0.76 for *N*_e_ = 100,000. This improvement does not continue with *N*_e_ being further increased to 1,000,000 (sum of squares = 10.16). Coalescent simulations with such high *N*_e_ overestimate the extent of incongruence, with no genes expected to support any of the clades in 80% of the simulated datasets.

MCcoal simulations were also run with the nuclear-only species tree providing the guide phylogeny. Here the aim was to determine whether ILS can potentially explain the mtDNA supporting *Macropus* monophyly or placing *M. irma* distantly from other members of *M. (Notamacropus)*. Effective population size was set to mitochondrial equivalency for the same populations (one quarter of the corresponding nuclear values). All simulated mtDNA sequences favoured the *Wallabia*/*M. (Notamacropus)* and *M. (Notamacropus)* groupings for each of the three lower *N*_e_ values (250, 2,500, 25,000). At *N*_e_ = 250,000 these fell to 46% and 66% respectively, although support among the simulated datasets for *Macropus* monophyly remained at 0% and only increased to 1.5% for *M. irma* falling within any grouping outside core-*Notomacropus* that is at least as shallow as *M. (Osphranter)* – where *M. irma* was placed on the observed mtDNA. Hence, the mitochondrial placements of *Wallabia* and *M. irma* are difficult to reconcile with ILS.

## Discussion

### Mitochondrial sequences provide confirmation and incongruence

Molecular studies have consistently shown that *Lagorchestes*, *Wallabia* and the *Macropus* subgenera *M. (Macropus)*, *M. (Osphranter)* and *M. (Notamacropus)* diverged from each other in rapid succession. We estimate that together, their consecutive divergences cover a temporal window of little more than 2 million years (Figure 1B, Table 3), in agreement with Meredith et al. [7]. The short internal branches may provide low phylogenetic resolution due to stochasticity associated with few substitutions along branches and conflicting signals attributable to incomplete lineage sorting (ILS) among genes. This expectation was borne out for the nuclear dataset, with all relationships among the five groups poorly resolved (Figure 2B, BP_ML_ from 36–59%).

Adding the mitochondrial (mt) sequences to the nuclear dataset substantially enhances resolution (Figure 4A). All groupings on the tree receive ≥90% BP_ML_ and are consistent with the supertree (Figure 1A) modified from Cardillo et al. [6], with the exception of a near-trichotomy among the *Macropus* subgenera. As strong as these results are, there is significant incongruence between the five individual nuclear genes (Figure S1). Moreover, mtDNA discordance with the combined nuclear sequences (see Figure 2) necessitates caution, especially for inferrig the affinities of the swamp wallaby (*W. bicolor*) and the black-gloved wallaby (*M. irma*).

### The relationship of *Wallabia* to *Macropus*

All concatenated and species tree analyses of the combined mtDNA and nuclear genes recover *W. bicolor* as sister to *Macropus* (Figure 4), consistent with recent morphological analyses [2], [12], [13]. If we accept this relationship, it implies that the nuclear (Nuc_17_) placement of *W. bicolor* within *Macropus* is an artefact, potentially of ILS among the individual genes. This interpretation is consistent with extreme incongruence among the nuclear genes on both MP and ML-based partition homogeneity tests (P≤0.005). Moreover, support for the overall nuclear placement with *M. (Notamacropus)* derives from only *BRCA1* and *vWF*, while *IRBP* favours the mtDNA placement as sister to *Macropus* (Table 2A).

We examined the incongruence further with coalescent simulations and show that with small populations the models overestimate support among the nuclear genes for clades within the diversification cluster. However, simulating deeper ILS consistent with *N*_e_ = 100,000 on the species tree closely matches observed support for these clades (Figure 3B). Neaves et al. [74] recently estimated *N*_e_ of similar magnitude for *M. fuliginosus*. If indeed *W. bicolor* does fall outside *Macropus*, then it might appear anomalous that only *IRBP* among the five nuclear genes favours *Macropus* monophyly. This result however, matches the coalescent simulations on the species tree under the best fitting *N*_e_; for 74% of the simulated datasets *Macropus* monophyly was recovered for only one (or none) of the nuclear genes. In contrast, none of the corresponding mtDNA coalescent simulations (Figure 3C) on the nuclear-only species tree favour *Macropus* monophyly. Hence, the observed mtDNA support for *Macropus* monophyly is unlikely to be an artefact of incomplete mitochondrial lineage sorting.

Strong mtDNA support for *Macropus* monophyly and apparently extensive ILS among nuclear loci within the diversification cluster around the base of *Macropus* caution against Meredith et al. 's [7] recommendation to subsume *Wallabia* within *Macropus*. Nevertheless, statistical support among our species tree analyses for excluding *W. bicolor* from *Macropus* is not conclusive. Indeed, coalescent simulations (Figure S2) on the species tree suggest that 30 or more nuclear loci may be required to confidently resolve relationships among the diversification cluster. However, considering our present phylogenetic results alongside the distinct browsing ecology and associated morphology/behaviour of *W. bicolor* [2], [4], [14], [75] and its unique 2n = 10(♀)/11(♂) karyotype, we believe that *Wallabia* currently warrants separate generic status.

### Deep mitochondrial introgression in *Macropus irma*

Incongruence between the mt and nuclear placements for the black-gloved wallaby (*M. irma*) differs in several respects from that concerning *W. bicolor*. It is the nuclear placement of *M. irma* with *M. (Notamacropus*) that concurs with morphology and none of the nuclear genes prefer the mt placement with *M. (Osphranter)* (Table 2). A further point of difference is that the concatenated (MtNuc_16_) analysis and two species tree reconstructions (MDC and BUCKy) support the nuclear placement for *M. irma*.

The mt placement of *M. irma* with wallaroos is difficult to reconcile with incomplete mitochondrial lineage sorting, being nested within *M. (Osphranter)* and because the path-length to its species tree position at the base of *M. (Notamacropus)* is 3–8 million years. Mitochondrial introgression may provide a more plausible explanation. Methods are being developed to directly test for introgressive hybridization, although these are not feasible without many loci or strong priors on the probability of hybridization [33], [34]. Nevertheless, our coalescent simulations (Figure 3C) further suggest that the aberrant mt placement of *M. irma* is not an artefact of incomplete lineage sorting. Even with the most extreme deep coalescence (ILS) scenario fewer than 2% of the simulated mtDNA datasets favoured *M. irma* falling outside its species tree grouping and into clades at least as shallow as *M. (Osphranter)*. Hence, on the available evidence the more likely explanation is that *M. irma* obtained its mt genome from introgressive hybridization with an ancestor of the wallaroos, the deepest such event yet hypothesised among marsupials.

Previous examples of hybridization or introgression among wild macropodids (from *Petrogale* and *Macropus*) involve closely related parent species [32], [76]. The absence of evidence for introgression among more distantly related macropodids may reflect sparse sampling. Certainly, captive-bred hybrids include more distantly related *Macropus* crosses as well as *Macropus*×*Wallabia* and reports of *Macropus*×*Thylogale* [77], [78]. The results of Neaves et al. [33] may also be relevant here. Despite finding evidence for introgression in 17 of 223 grey kangaroos in the *M. giganteus/M. fuliginosus* sympatry zone, no F1 individuals were identified. The authors interpreted this result as suggesting a role for selection in accelerating introgression of some loci into the gene pool at well beyond the actual rate of hybridization.

Adaptive introgression of nuclear loci, as proposed for the grey kangaroos has also been suggested for mitochondria in wild goats [79] and Hares [80]. It is also possible that introgression of *M. (Osphranter)* genes into *M. irma* has adaptive significance and may not be limited to mtDNA. Most notably, Milne and O'Higgins [81] found that skull shape principle components that were correlated with vegetation cropping and mastication grouped *M. irma* within *M. (Osphranter)*, thus matching the mtDNA and further suggesting adaptive convergence. This is consistent with Christensen [82] regarding *M. irma* as somewhat transitional in diet and locomotion between *M. (Notamacropus)* wallabies and the larger wallaroos and kangaroos of *M. (Osphranter)* and *M. (Macropus)*. In particular, *M. irma* favours more open habitats [82] and may rely on grazing and poorer quality plant material more than most of its wallaby relatives [83].

If *W. bicolor* is sister to *Macropus*, then sharing deep gene coalescences with the more ecologically similar *M. (Notamacropus)* wallabies rather than with the larger, grazing *M. (Macropus)* and *M. (Osphranter)* might also point towards adaptive significance. The influence of selection on patterns of ILS and introgression is poorly understood at present and clarification of our intimations concerning *M. irma* and *W. bicolor* requires more thorough genomic sampling and analysis of functional correlations.

### Variation among species tree inferences

Each of the MtNuc_16_ concatenated and species tree analyses recovered the same kangaroo phylogeny (Figure 4A–C), except for *BEAST placing *M. irma* as sister to *M. (Osphranter)*, close to the mtDNA relationship. One shortfall in the present usage of *BEAST is that species were represented by only one individual and therefore *N*_e_ could only be estimated for internal branches. It is not clear however that this should have any specific impact on the placement of *M. irma*. Indeed, running the analysis without the sequence data indicates that the placement of this taxon was not attributable to any aspect of the tree prior. Furthermore, using single individuals did not promote any other topological differences from the concatenated tree. Instead, the *BEAST result is consistent with mitochondrial introgression being the source of the incongruence concerning *M. irma*. This violates the assumption of *BEAST that ILS is the only source of incongruence. In contrast, another species tree method, BUCKy does not assume any particular source of incongruence and recovered the expected placement of *M. irma* as sister to core-*Notamacropus*.

It is interesting that MDC recovered the expected placement for *M. irma*, despite also assuming that incongruence derives solely from ILS. The explanation may lie in MDC being a consensus method, such that no matter how strong the signal from the mtDNA, it will be overwhelmed by consistent signal among multiple independent nuclear loci. *BEAST is not a consensus method. Instead it models the multi-species coalescent to co-infer gene trees embedded in a species tree, which as Heled and Drummond [27] explain, effectively provides a “reverse auction”, where the lowest bidder can set the limit. Consequently, *BEAST is a powerful tool for identifying true species relationships and divergence times when incongruence derives from ILS. However, the reverse auction might often leave *BEAST less robust than consensus methods to introgression or paralogy. Nevertheless, we believe that multi-species coalescent methods such as *BEAST are an important advance for phylogenetics. Allowing for limited post-speciation gene flow [84] will improve their reliability and will provide a valuable test for distinguishing incongruence from ILS.

Our partition homogeneity testing and analysis of coalescent simulations on the species tree (Figure 3B) are consistent with widespread ILS across the cluster of six rapidly diverged lineages, Dendrolagini, *Lagorchestes*, *Wallabia* and the three *Macropus* subgenera. The apparent introgression of *M. (Osphranter)* mtDNA into *M. irma* is the sole instance of significant mitochondrial discord with the species tree. These patterns of incongruence, although too few to draw strong conclusions on, nevertheless fit the expectations set out earlier. Specifically, that ILS will be more common among nuclear loci, consistent with longer coalescence times than for mtDNA, which in turn will be more susceptible to introgressive selective sweeps associated with fitness differences across populations, promoted by Muller's ratchet.

Overall our results suggest that sampling multiple mt genes is well suited to providing a first estimate for species-level phylogenies among marsupials and in combination with the five nuclear genes, substantially enhances phylogenetic resolution. Moreover, concerns that mt signal from three-fold as many parsimony-informative characters would swamp nuclear signal were unfounded. Combined analyses generally favoured the nuclear placements for *M. irma* and among the *Macropus* subgenera over their mt placements. The combined data only favoured the mt placement of *Wallabia*, for which the nuclear loci themselves were incongruent and contradicted morphology. However, larger scale nuclear genomic sampling will ultimately provide a more comprehensive understanding of evolutionary history, including for whether selective advantages contribute to patterns of ILS and introgression in *W. bicolor* and *M. irma*.

## Supporting Information

Figure S1 **Individual nuclear gene phylogenies.** (A) *BRCA1*, (B*) IRBP*, (C) *ApoB*, (D) *vWF* and (E) *RAG1*. MrBayes 3.1.2 Bayesian posterior probabilities (above 0.5) and PAUP* 4.0b10 maximum likelihood bootstrap percentages (>50) are shown above and below branches respectively. Analyses were carried out as per the primary analysis for Nuc_17_.
(PDF)

Figure S2 **Maximum likelihood bootstrap identification of the number of genes required to resolve macropodid phylogeny.** (A) for *Macropus* monophyly and (B) for the *M. (Macropus)-M. (Osphranter)* grouping. Simulated gene sequences (1,000 bp) were added in increments of five. Ten independent runs were continued until sufficient sequences were added for ML_BP_ >95%. Seven of 10 simulations reached 95% ML_BP_ with 20 genes for *Macropus* and 35 genes for *M. (Macropus)-M. (Osphranter)*.
(PDF)

Table S1 **List of primers and conditions used for amplifying macropodoid DNA.**
(PDF)

Table S2 **GenBank accession numbers for the sequences included in the mitochondrial data matrices.**
(PDF)

Table S3 **jModelTest selections for mitochondrial and nuclear data partitions.**
(PDF)
