# Supplementary material for: Inferring Kangaroo Phylogeny from Incongruent Nuclear and Mitochondrial Genes
Source: PLoS One. 2013 Feb 22;8(2):e57745. doi: 10.1371/journal.pone.0057745 (PMC3579791; doi:10.1371/journal.pone.0057745)

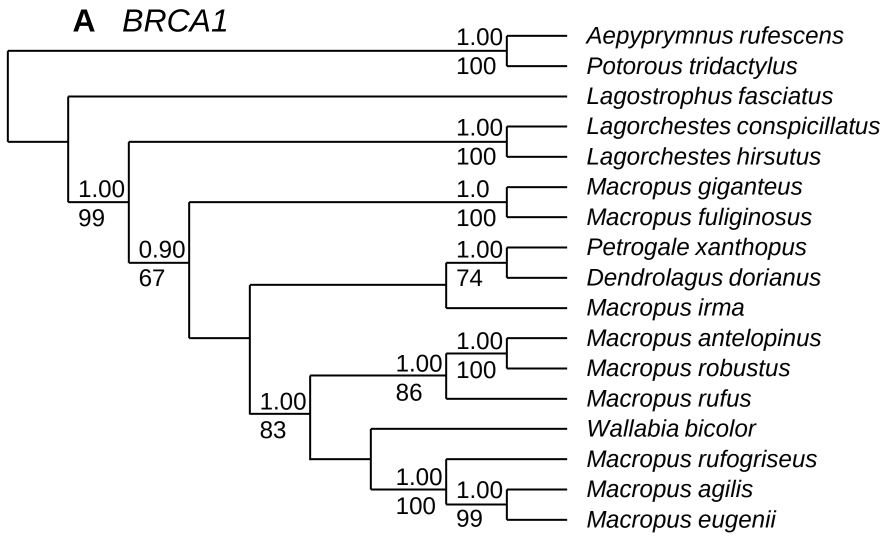

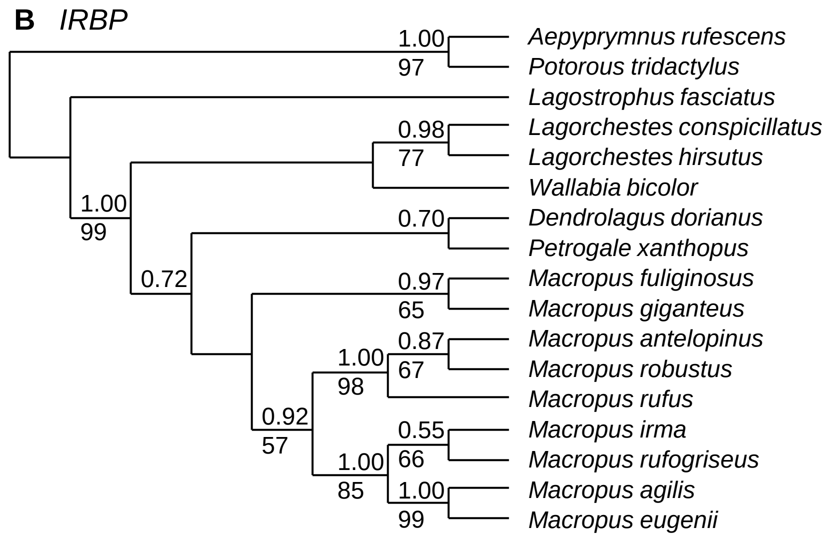

Figure S1. **Individual nuclear gene phylogenies**. (A) *BRCA1*, (B*) IRBP*, (C) *ApoB*, (D) *vWF* and (E) *RAG1*. MrBayes 3.2 Bayesian posterior probabilities (above 0.5) and PAUP* 4.0b10 maximum likelihood bootstrap percentages (>50) are shown above and below branches respectively. Analyses were carried out as per the primary analysis for Nuc_{17}, with substitution models shown in Table 2.

Figure S1 - continued

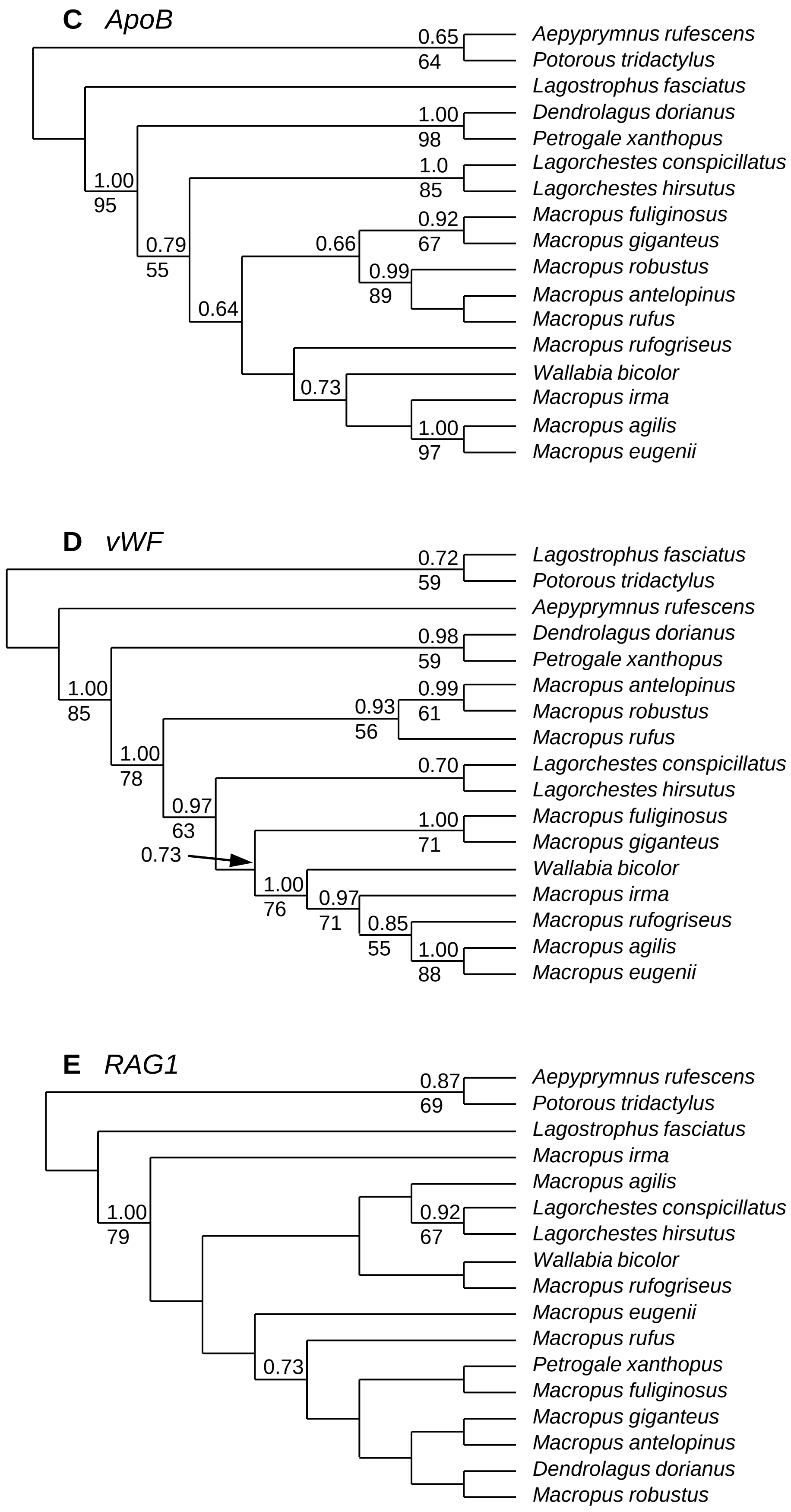

Supplement: Figure S1 — Individual nuclear gene phylogenies. (A) BRCA1, (B) IRBP, (C) ApoB, (D) vWF and (E) RAG1. MrBayes 3.1.2 Bayesian posterior probabilities (above 0.5) and PAUP* 4.0b10 maximum likelihood bootstrap percentages (>50) are shown above and below branches respectively. Analyses were carried out as per the primary analysis for Nuc17. (PDF) [file pone.0057745.s001.pdf]
