# Supplementary material for: Inferring Kangaroo Phylogeny from Incongruent Nuclear and Mitochondrial Genes
Source: PLoS One. 2013 Feb 22;8(2):e57745. doi: 10.1371/journal.pone.0057745 (PMC3579791; doi:10.1371/journal.pone.0057745)
Supplement: Figure S2 — Maximum likelihood bootstrap identification of the number of genes required to resolve macropodid phylogeny. (A) for Macropus monophyly and (B) for the M. (Macropus)-M. (Osphranter) grouping. Simulated gene sequences (1,000 bp) were added in increments of five. Ten independent runs were continued until sufficient sequences were added for MLBP >95%. Seven of 10 simulations reached 95% MLBP with 20 genes for Macropus and 35 genes for M. (Macropus)-M. (Osphranter). (PDF) [file pone.0057745.s002.pdf]

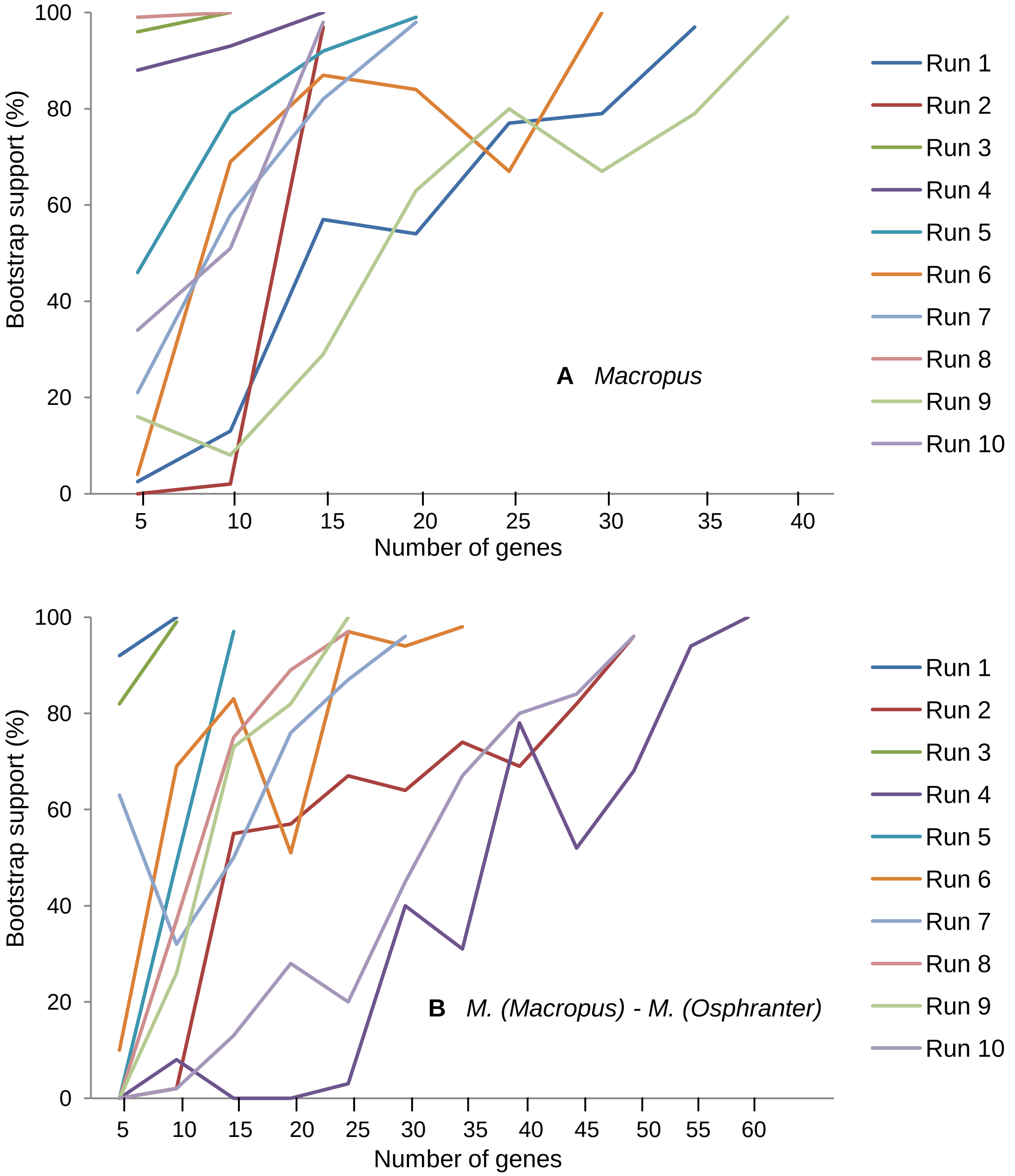

Figure S2. **Maximum likelihood bootstrap identification of the number of genes required to resolve macropodid phylogeny**. (A) for *Macropus* monophyly and (B) for the *M. (Macropus)-M. (Osphranter)* grouping. Simulated gene sequences (1,000 bp) were added in increments of five. Ten independent runs were continued until sufficient sequences were added for ML_{BP} >95%. Seven of 10 simulations reached 95% ML_{BP} with 20 genes for *Macropus* and 35 genes for *M. (Macropus)-M. (Osphranter)*.

Gene sequences were simulated on the overall species tree as described in the Methods. Coalescent trees were simulated in MCcoal with the best fitting N_e (100,000) from the individual gene simulations (Figure 3). These trees were used as guides for simulating sequences in Seq-Gen1.3.2 under the GTR+I+Γ model inferred from the Nuc_{16} concatenate. ML bootstrap on the concatenated simulated datasets was carried out in PAUP*4.0b10. Other coalescent simulations, either with two individuals per species or on the nuclear-only species tree produced similar results.
