# Supplementary material for: Inferring Kangaroo Phylogeny from Incongruent Nuclear and Mitochondrial Genes
Source: PLoS One. 2013 Feb 22;8(2):e57745. doi: 10.1371/journal.pone.0057745 (PMC3579791; doi:10.1371/journal.pone.0057745)
Supplement: Table S1 — List of primers and conditions used for amplifying macropodoid DNA. (PDF) [file pone.0057745.s003.pdf]

Table S1. List of primers and conditions used for amplifying macropodid DNA

Genome	Gene amplified[1]	Primer name	Sequence (5' – 3')	~ Amplicon Length (bp)	Annealing temp. (°C)	Primer reference
Nuclear	*IRBP*	IR_A_F<br>IR_B_R	ATGGCCAAGGTCCTCTTGGATAACTACTGCTT<br>AGGTTCCATGATGAGGTGCTCCGTGTCCTG	1300	58[2]	Meredith et al. 2008
	IRBP	IR_A_F<br>IR_N_R	ATGGCCAAGGTCCTCTTGGATAACTACTGCTT<br>CGCATCTTCTTGAGGATGTAG	600	62[2]	Meredith et al. 2008
	IRBP	IR_M_F<br>IR_B_R	CCCSTTTGTCATTTCCTACC<br>AGGTTCCATGATGAGGTGCTCCGTGTCCTG	700	58[2]	Meredith et al. 2008
	ApoB	F60<br>MARS_R820	GGAGAAGCCAAYCTGGATTTCYT<br>GTYGTCCCATCTAACTTATACTGC	750	60[2]	Meredith et al. 2008
	ApoB	F60<br>MARS_R474	GGAGAAGCCAAYCTGGATTTCYT<br>CATGTAGCCAAWTGRTGGCATCTC	500	60[2]	Meredith et al. 2008
	ApoB	MARS_F420<br>MARS_R820	CAATTCCTGAAATGACTCTGCC<br>GTYGTCCCATCTAACTTATACTGC	450	60[2]	Meredith et al. 2008
Mitochondrial	NADH1	ND1_1F<br>ND1_1R	GGCAGAGCTGGYAATTGCAT<br>GAAATAAGGGGGCTTDCAC	960	55[3,4]	Cao et al. 1998
	NADH2	tMetFor<br>tAlaRev	ATACCCCGAAAATGTTGGTTC<br>GATTTGCGTTCGAKTGATGCAAG	1050	55[3,4]	Osborne & Christidis, 2001
	Cyt*b*	Cyt*b*_F14195<br>Cyt*b*_R15472	CATTTTAGTATGGACTCTAACCATAACC<br>AGGGTGTTATACCTTCATTTTGG	1150	54[4]	This study
	12S rRNA	L2180_F<br>M_12S6_R	AAAGCACAGCACTGAAGATGC<br>GCTCAAAATGGTCAGGGTTAGCTG	950	58[4]	mod. from Titus & Frost 1996<br>This study
	16S rRNA	M_12S3_F<br>M_12S5_R	AAACGTTAGGTCAAGGTGTAGC<br>GCAATTGCCAGCYCTGCCAC	1950	58[4]	This study
	12/16S rRNA	L2180<br>H3628	AAAGCACAGCACTGAAGATGCTG<br>GCTGTCTTTACAGGTGGCTGCT	1560	55[3]	mod. from Titus & Frost 1996<br>Reed et al. 2001

16S rRNA	16Sar	CGCCTGTTTATCAAAAACAT	550	55[3]	Palumbi et al. 1996
	16Sbr	CCGGTYTGAACTCAGATCAYGT			Palumbi et al. 1996
NADH2	ND2a_3F	GGGGTGMAAGCCCCCTTATT	1205	55[3]	This study
	tAlaRev	GATTTGCGTTCGAKTGATGCAAG			
NADH1	ND1_1F	GGCAGAGCTGGYAATTGCAT	960	55[3]	Cao et al. 1998
	ND1_1R	GAAATAAGGGGGCTTDCAC			

[1] Post-PCR purifications were performed either using Shrimp Alkaline Phosphatase and Exonuclease I (GE Healthcare/Crown scientific) or alternatively, using a Qiaquick purification kit (Qiagen Sciences, MD, USA). All amplicons were sequenced by Macrogen (Seoul, South Korea) on an Applied Biosystems 3730XL capillary sequencer. Geneious 5.4.3 (Biomatters, NZ) was used to examine the chromatograms and assemble the forward and reverse contigs for each gene.

[2] Murdoch University, nuclear genes: initial denaturation (5 min at 94°C); 35 cycles of denaturation (45 sec at 94°C); variable annealing temperatures (see Table above, for 45 sec); extension (68°C for 1-2 min, depending on fragment length); final extension (10 min at 72°C).

[3] Australian National University, mitochondrial genes: initial denaturation (2 min at 94 C); 30 cycles of denaturation (15 sec at 94 C), annealing (30 sec at 50 – 55 C), extension (90 sec at 68 C); final extension (4 min at 68C).

[4] Murdoch University, mitochondrial genes: initial denaturation (3 min at 94°C); 35 cycles of denaturation (30 sec at 94°C); variable annealing temperatures (see Table above, for 30 sec); extension (90 sec at 68°C); final extension (10 min at 72°C).

References

Cao Y, Janke A, Waddell PJ, Westerman M, Takenaka O, Murata S, Okada N, Pääbo S, Hasegawa M (1998). Conflict among individual mitochondrial proteins in resolving the phylogeny of eutherian orders. J. Mol. Evol. 47: 307-322.

Meredith RW, Westerman M, Springer MS (2008). A timescale and phylogeny for "bandicoots" (Peramelemorphia: Marsupialia) based on sequences for five nuclear genes. Mol. Phylogenet. Evol. 47: 1-20.

Osborne ML, Christidis L, 2001. Molecular Phylogenetics of Australo–Papuan Possums and Gliders (Family Petauridae). Mol. Phylogenet. Evol. 20: 211–224.

Palumbi S, Martin A, Romano S, McMillan WO, Stice L, Grabowski G (1991). The simple fool’s guide to PCR, Ver. 2.0 Honolulu, University of Hawaii.

Reed K, Keogh JS., Scott IA, Roberts JD, Doughty P (2001). Molecular phylogeny of the Australian frog genera Crinia, Geocrinia, and allied taxa (Anura: Myobatrichidae). Mol. Phylogent. Evol. 21: 294-308.

Titus TA, Frost DR (1996). Molecular homology assessment and phylogeny in the lizard family Opluridae (Squamata: Iguania), Mol. Phylogent. Evol. 6: 49-62.
