# Supplementary material for: Inferring Kangaroo Phylogeny from Incongruent Nuclear and Mitochondrial Genes
Source: PLoS One. 2013 Feb 22;8(2):e57745. doi: 10.1371/journal.pone.0057745 (PMC3579791; doi:10.1371/journal.pone.0057745)
Supplement: Table S2 — GenBank accession numbers for the sequences included in the mitochondrial data matrices. (PDF) [file pone.0057745.s004.pdf]

Table S2. GenBank accession numbers for sequences included in the mitochondrial data matrices.

Taxon	*NADH1& 2*	*Cytb*	12S&16S rRNA
Aeppyprymnus rufescens	JN003376, JN003377	EU086684	AF027999
Potorous tridactylus	AJ639873	AJ639873	AJ639873
Lagostrophus fasciatus	NC008447	NC008447	NC008447
Dendrolagus dorianus	JN003378, JN003379	JN003380	AF027989
Petrogale xanthopus	JN003381, JN003382	EF368032	AF187886
Lagorchestes hirsutus	NC008136	NC008136	NC008136
Lagorchestes conspicillatus	JN003383, JN003384	JN003385	JN003386
Wallabia bicolor	JN003387, JN003388	EF368031, JX504693	AF027987
Macropus irma	JN967004, JN967006	JN967002	JN966998, JN967000
Macropus rufogriseus	JN003392, JN003393	EF368027	JN003394, JN003395
Macropus eugenii	JN003389, JN003390	AY237226	AY245606, JN003391
Macropus agilis	AF425981	EF368029	AF027986
Macropus dorsalis	JN00402, JN003403		JN003404
Macropus rufus	JN967005, JN967007	JN967003	JN966999, JN967001
Macropus robustus	Y10524	Y10524	Y10524
Macropus antilopinus		EF368024	
Macropus giganteus	JN003396, JN003397	U87137	AF187885
Macropus fuliginosus	JN003398, JN003399	JN003400	JN003401

New sequences obtained in the present study are underlined.
