# Supplementary material for: Inferring Kangaroo Phylogeny from Incongruent Nuclear and Mitochondrial Genes
Source: PLoS One. 2013 Feb 22;8(2):e57745. doi: 10.1371/journal.pone.0057745 (PMC3579791; doi:10.1371/journal.pone.0057745)
Supplement: Table S3 — jModelTest selections for mitochondrial and nuclear data partitions. (PDF) [file pone.0057745.s005.pdf]

Table S3. jModelTest selections for mitochondrial and nuclear data partitions.

Mitochondrial sequences		Nuclear sequences	
Partition[1]	Model[2]	Partition	Model[2]
Protein codons		*Protein codons*	
1st codon positions	GTR+I+ Γ	1st codon positions	TIM+ Γ
2nd codon positions	K81uf+I+ Γ	2nd codon positions	TVM+ Γ
3rd codon positions	TIM+ Γ	3rd codon positions	TVMef+ Γ
RNA structure		*Genes*	
Stem positions	SYM+I+ Γ	*BRCA1*	TrN+Γ
Loop positions	TIM+I+ Γ	*ApoB*	TrN+Γ
		IRBP	TVM+Γ
		RAG1	HKY+I+ Γ
		vWF	K80+I+ Γ

[1] Meredith et al. (2008) modelled the nuclear data as five gene-wise partitions, whereas we employ three codon-wise partitions. Both partitioning schemes identified the same ML tree topology for Nuc_{17} (Figure 1B). However codon-wise partitioning provides higher likelihood (in PAUP*) with fewer free parameters (–13,107.338, df=113) than gene-wise partitioning (–13,115.969, df=184). Partitioning by both gene and codon resulted in several partitions with far more parameters requiring estimation than variable sites and hence, was considered too parameter rich.

[2] The most general of either the hierarchical likelihood ratio test or Akaike Information Criterion model suggestions is reported.

Reference

Meredith RW, Westerman M, Springer MS (2008) A phylogeny and timescale for the living genera of kangaroos and kin (Macropodiformes: Marsupialia) based on nuclear DNA sequences. Aust J Zool 56: 395-410.
